# Editorial: Hypoxia in cardiovascular disease

**DOI:** 10.3389/fcvm.2022.990013

**Published:** 2022-08-05

**Authors:** Grzegorz Bilo, Hannes Gatterer, Camilla Torlasco, Francisco C. Villafuerte, Gianfranco Parati

**Affiliations:** ^1^Department of Cardiology, San Luca Hospital, Istituto Auxologico Italiano, IRCCS, Milan, Italy; ^2^Department of Medicine and Surgery, University of Milano-Bicocca, Milan, Italy; ^3^Eurac Research, Institute of Mountain Emergency Medicine, Bolzano, Italy; ^4^Laboratorio de Fisiología Comparada/Laboratorio de Fisiología del Transporte de Oxígeno, Facultad de Ciencias y Filosofía, Universidad Peruana Cayetano Heredia, Lima, Peru

**Keywords:** hypoxia, high altitude, cardiovascular risk, hypertension, thrombosis

Hypoxia occurs in both physiological (breathing air with reduced oxygen content, e.g., at high altitude) and pathological conditions (e.g., respiratory diseases, heart failure, sleep apnea). It can be acute or chronic, continuous or intermittent, generalized or localized, hypo- or normobaric. Hypoxia is a potent modifier of cardiovascular function, its effects being mediated on a molecular level largely by hypoxia inducible factors (HIFs). At the systemic level, key mechanisms involved in cardiovascular responses to hypoxia include changes in autonomic nervous system activity mediated by chemoreflexes, directly induced changes in vascular tone (peripheral vasodilation and pulmonary vasoconstriction), as well as humoral changes, for example suppression of the renin-angiotensin-aldosterone system (1, 2). These responses are largely aimed at counteracting reduced oxygen availability, but may also become maladaptive, for instance in the case of high-altitude pulmonary or cerebral edema.

From the clinical point of view, hypoxia exposure has different implications: it may modify the risk of cardiovascular complications through its influence on cardiac and vascular function, autonomic nervous system, fluid homeostasis and coagulation; and it may also modify the effects of cardiovascular therapies (3). Controlled hypoxia may represent a treatment modality, as in the case of interval hypoxic training. Despite the fact that highly prevalent cardiovascular diseases frequently coexist with hypoxia, there are still large areas of uncertainty in this regard (1).

The present Research Topic aimed to provide further insight into the pathophysiological mechanisms linking hypoxia to cardiovascular disease, into clinical and epidemiological implications of this relationship and into the related therapeutic options. The included papers encompass several aspects, from molecular mechanisms to clinical implications.

A comprehensive review illustrating the link between hypoxia and cardiovascular disease was provided by Garcia Rojas et al.. They described how hypoxia plays a central role in the pathogenesis and pathophysiology of all cardiovascular diseases, and how the organism reacts to cope with reduced oxygen availability. Compensatory mechanisms are mostly mediated by the hypoxia inducible factors (HIFs), and affect erythropoiesis, glucose transport, angiogenesis, glycolytic metabolism, handling of reactive oxygen species, cell proliferation and survival, among others. The global body response to hypoxia depends both on the severity of hypoxia and its duration. On this basis, studying the body response to high-altitude hypobaric hypoxia can help understanding these mechanisms, leading to more effective treatment strategies with the final goal to reduce morbidity and mortality (Garcia Rojas et al.).

Local tissue hypoxia is the foremost pathophysiological factor implied in the ischemic cardiovascular conditions, in particular ischemic heart disease. A better understanding of hypoxia-mediated molecular mechanisms of myocardial ischemia may have profound consequences in developing better cardioprotective therapies. These aspects were investigated in two papers included in this Research Topic.

Chen et al. established an isolated cardiomyocyte model of hypoxic/reoxygenation injury to study whether the protective effect of diazoxide post-conditioning on myocardial ischemic-reperfusion injury is related to the HIF-1/HRE pathway and whether reactive oxygen species (ROS) play a role in this process. The authors report that diazoxide post-conditioning promotes opening of the mitoK_ATP_ channel to generate a moderate ROS level that activates the HIF-1/HRE pathway and subsequently induces myocardial protection (Chen et al.). The results provide mechanistic insights into the postconditioning effects mediated by diazoxide; it is important to mention here that diazoxide administration may exert multiple effects in the living organism (e.g., provision of glucose, vasodilation, action on cardiac sarcolemmal K_ATP_ channel) that may also play a role in the cardioprotective properties (4). The therapeutic potential of diazoxide is, however, limited by numerous side effects including shortness of breath, swelling in extremities, tachycardia, chest pain, blurred vision, bruising or bleeding, unusual weakness and decreased frequency of urination (4).

Cardiac fibrosis is among the principal pathological mechanisms involved in heart failure (5), and local hypoxia induced by myocardial ischemia may play a role in this regard. While appropriate fibrotic response contributes to tissue repair, excessive fibrosis caused by continuous activation of myofibroblasts can lead to a gradual decrease in tissue compliance, reduced nutrient and oxygen delivery, and increased myocardial atrophy and cell death, leading to progressive left ventricular dilation and dysfunction (6). A disintegrin and metalloproteinase with thrombospondin motifs (ADAMTS) family proteins are secreted proteases that bind to the extracellular matrix, and certain ADAMTSs play an important role in regulating cell proliferation, adhesion, migration and intracellular signal transduction (7). In this Research Topic, Zha et al. reported the results of a series of *in vitro* and *in vivo* experiments on the role of ADAMTS8 in myocardial fibrosis after injury or stress. Myocardial infarction (MI), cardiac fibrosis induced by transverse aortic constriction, and heart failure, all caused an increase in ADAMTS8 expression, and were accompanied by myofibroblast activation and collagen fiber deposition. Epidermal growth factor (EGFR) and related downstream signaling pathways were partially activated by increased ADAMTS8 secretion, thereby promoting fibroblast activation *in vitro*. Also, ADAMTS8 overexpression *in vivo* impaired cardiac function and promoted myocardial fibrosis in the MI rat model. Interestingly, hypoxia increased ADAMTS8 expression in cardiomyocytes more significantly than in cardiac fibroblasts, implying an important role of the former in promoting fibrosis. Lastly, Zha et al. showed that pharmacological reduction of ADAMTS8 expression in cardiac myocytes and fibroblasts by mebendazole ameliorated heart fibrosis.

Thrombosis is one of the key elements of cardiovascular pathophysiology. However, the role of hypoxia in the thrombogenesis is not well established. In the present Research Topic Treml et al. provided a systematic review of the available evidence in this field, focusing on the influence of environmental (high altitude) hypoxia on coagulation, fibrinolysis, and platelet function. The careful overview of 20 included studies led to the conclusion that, while some changes may occur in both pro- and antithrombotic mechanisms, the overall balance between coagulation on one side and decreased platelet activity and increased fibrinolysis on the other, remains largely unchanged, at least in healthy individuals. This evidence, however, cannot be regarded as conclusive, considering high heterogeneity of available studies and the presence of numerous confounding factors (Treml et al.).

Finally, the clinical paper by Lang et al. addressed a relevant issue related to high-altitude hypoxia, in its particular model known as Chronic Intermittent Hypoxia (CIH). This condition is characterized by an alternance of several days' exposure to hypoxia and to normoxia and occurs in individuals (mainly miners) who work at high altitude according to such a particular shift model. In this paper blood pressure (BP) responses in both normotensive and hypertensive Chilean miners were assessed both at high and low altitude, by means of conventional measurements and ambulatory blood pressure monitoring (Lang et al.). It was observed that, even though the participants had been exposed to CIH since at least 2 years, BP increase during acute exposure to hypobaric hypoxia was present. Such response is similar to that observed in lowlanders with no previous hypoxia exposure (2, 8), although in miners blood pressure normalization appeared to be faster (conventional BP fully normalized after 7 days of exposure). Importantly, in hypertensive participants with well-controlled conventional BP at sea level, BP at high altitude was only mildly increased, indicating good efficacy of antihypertensive treatment in this condition. Moreover, a considerable number of participants (including those without previous hypertension diagnosis) presented with masked hypertension (elevated ambulatory BP with normal BP on conventional measurements), indicating the importance of out-of-office BP measurements in this setting (Lang et al.).

Overall, we believe that the “Hypoxia in Cardiovascular Disease” Research Topic achieved its aims by providing a relevant contribution in various aspects of this field, although many important issues remain to be addressed and better understood, in particular in the therapeutic area.

## Author contributions

GB, HG, CT, and FV drafted parts of the manuscript and reviewed the entire manuscript draft. GP reviewed the entire manuscript draft. All authors contributed to the article and approved the submitted version.

## Conflict of interest

The authors declare that the research was conducted in the absence of any commercial or financial relationships that could be construed as a potential conflict of interest.

## Publisher's note

All claims expressed in this article are solely those of the authors and do not necessarily represent those of their affiliated organizations, or those of the publisher, the editors and the reviewers. Any product that may be evaluated in this article, or claim that may be made by its manufacturer, is not guaranteed or endorsed by the publisher.
